# Automated Filling Equipment Allows Increase in the Maximum Dose to Be Filled in the Cyclops^®^ High Dose Dry Powder Inhalation Device While Maintaining Dispersibility

**DOI:** 10.3390/pharmaceutics12070645

**Published:** 2020-07-09

**Authors:** Imco Sibum, Paul Hagedoorn, Carel O. Botterman, Henderik W. Frijlink, Floris Grasmeijer

**Affiliations:** Department of Pharmaceutical Technology and Biopharmacy, Faculty of Science and Engineering, University of Groningen, 9700 AB Groningen, The Netherlands; p.hagedoorn@rug.nl (P.H.); c.o.botterman@student.rug.nl (C.O.B.); h.w.frijlink@rug.nl (H.W.F.); f.grasmeijer@rug.nl (F.G.)

**Keywords:** high dose pulmonary delivery, dry powder inhaler, automatic filling, vacuum drum filler, inhalation, tuberculosis

## Abstract

In recent years there has been increasing interest in the pulmonary delivery of high dose dry powder drugs, such as antibiotics. Drugs in this class need to be dosed in doses far over 2.5 mg, and the use of excipients should therefore be minimized. To our knowledge, the effect of the automatic filling of high dose drug formulations on the maximum dose that can be filled in powder inhalers, and on the dispersion behavior of the powder, have not been described so far. In this study, we aimed to investigate these effects after filling with an Omnidose, a vacuum drum filler. Furthermore, the precision and accuracy of the filling process were investigated. Two formulations were used—an isoniazid formulation we reported previously and an amikacin formulation. Both formulations could be precisely and accurately dosed in a vacuum pressure range of 200 to 600 mbar. No change in dispersion was seen after automatic filling. Retention was decreased, with an optimum vacuum pressure range found from 400 to 600 mbar. The nominal dose for amikacin was 57 mg, which resulted in a fine particle dose of 47.26 ± 1.72 mg. The nominal dose for isoniazid could be increased to 150 mg, resulting in a fine particle dose of 107.35 ± 13.52 mg. These findings may contribute to the understanding of the upscaling of high dose dry powder inhalation products.

## 1. Introduction

For about 50 years, the pulmonary administration of highly potent low dose drugs, in the form of dry powders, has been used to treat pulmonary diseases [1]. Dry powder particles in the aerosol for inhalation should have a size between 1 and 5 µm to obtain adequate lung deposition [2]. This size requirement of the powder negatively affects its bulk flow properties [1,3]. To improve the flow properties, and with that dose reproducibility, low dose drugs are usually formulated in adhesive mixtures, where the drug in question adheres to the surface of freely flowing coarse lactose carrier particles [4,5].

In recent years there has been increasing interest in the pulmonary administration of low potency, high dose drugs, such as antibiotics. Drugs in this class need to be dosed in doses over 2.5 mg without excipients [1]. In reality, most of the drugs in this group need to be dosed much higher than that. However, at doses above 2.5 mg, improving the flow properties, and thus dose reproducibility, by formulating them in adhesive mixtures is no longer feasible because this formulation type usually does not contain more than 4% active drug [1].

The automatic filling of low dose formulations has been extensively investigated. Multiple filling techniques exist, such as “pepper-shaker” filling, tamp filling, vacuum drum filling, vacuum dosator filling, and dosator nozzle filling. However, almost all of them are designed to work with free-flowing adhesive mixtures [6,7,8,9,10,11,12]. To our knowledge there is no literature available that describes the automatic filling of high dose drugs which contain no or only minor amounts of excipient. As the physicochemical properties of the formulation are not masked by lactose carrier particles, automatic dosing in a reproducible way is a significant challenge. Of course, non-reproducible filling results in an inconsistent dose being delivered to the patient, which is undesirable. Moreover, it is unknown what the effect of the automatic filling on the maximum dose and dispersion behaviors are.

Not only do the physicochemical properties of the formulation have an effect on automatic dosing, automatic dosing may also have an effect on the formulation and its performance. Compaction, pelletization, or the formation of aggregates may occur by design or as a byproduct of automatic filling [6]. This is likely to have an effect on the dispersion behavior of the formulation. The poor break up of aggregates may lower the fine particle fraction (FPF), whereas, on the other hand, increased disintegration forces (e.g., through impaction) may increase the FPF. Additionally, powder may be further compacted in the dosing system of the inhaler, which lowers powder emission from the inhaler. This introduces other causes of inconsistent dose delivery to the patient.

**Figure 1:**
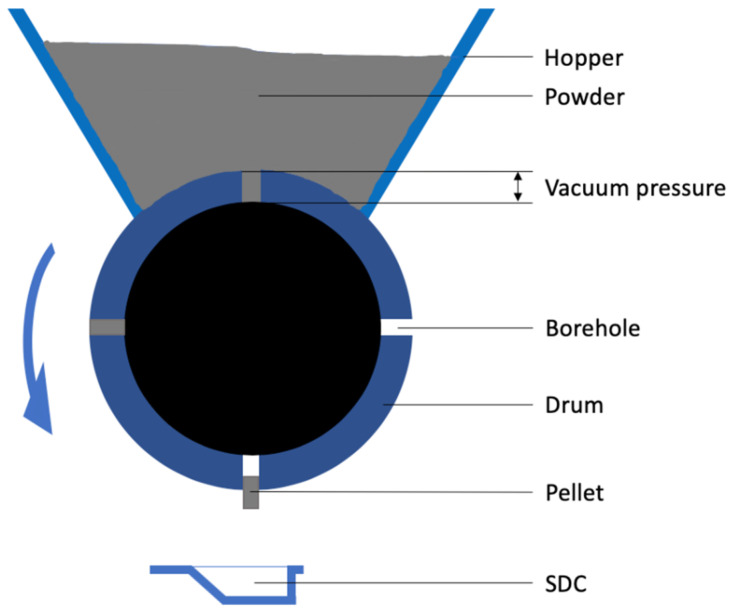
A schematic representation of the vacuum drum filler used in this study.

In this study, the Omnidose powder filling machine is used to automatically fill single dose cartridges (SDC) that are used in the Twincer^®^ and Cyclops^®^ dry powder inhalers (DPI). The Omnidose is a vacuum drum filler [6], where boreholes in a drum are filled with powder from a hopper driven by a difference in air pressure, called vacuum pressure in this article (Figure 1). In this way, powder plugs or pellets are formed with the shape and volume of the boreholes, which are then dropped in the SDCs. The density and, therefore, the mass of each individual pellet depends on the vacuum pressure applied.

To determine if the Omnidose can precisely and accurately dose high dose drugs, we performed experiments with two spray-dried formulations—an amikacin formulation that contains 1% L-leucine and an extensively characterized isoniazid formulation that contains 3% trileucine [13,14]. Both are antibiotics used in the treatment of tuberculosis [15]. A substantial amount of SDCs were filled under different conditions, after which the uniformity of dosage units was calculated. Furthermore, the effect on the dispersion of the powders from the Cyclops^®^ DPI was determined and compared to hand-filled SDCs.

## 2. Materials and Methods

### 2.1. Materials

Isoniazid and L-leucine used for this study were purchased from Sigma Aldrich (St. Louis, MO, United States). Trileucine was purchased from Bachem (Bubendorf, Switzerland). Amikacin was obtained from OfiPharma (Ter Apel, The Netherlands).

### 2.2. Spray-Drying

Isoniazid and amikacin powders were spray-dried using a B-290 mini spray drier, obtained from Büchi (Flawil, Switzerland). To obtain enough powder for automatic filling, isoniazid was spray-dried in three sessions and amikacin in two. The difference being explained by the fact that the process speed of the isoniazid formulation is lower. Isoniazid was spray-dried with 3% (*w/w*) trileucine in demineralized water, with an inlet temperature of 40 °C and a feed rate of 1 mL/min. The amikacin solutions in demineralized water contained 1% (*w/w*) L-leucine and were spray dried at 130 °C and 2.5 mL/min. A NE-300 syringe pump, obtained from ProSense B.V. (Oosterhout, The Netherlands) was used to control the feed rate. Other conditions were kept the same for both compounds. The other conditions for the two compounds were an atomizing airflow (Qflow) of 50 mm, an aspirator air flow of 100%, and a total solute concentration of 50 mg/mL.

### 2.3. Primary Particle Size Distribution Analysis

The primary particle size distributions of the produced powders were measured by laser diffraction analysis. A HELOS diffraction unit equipped with a RODOS dry disperser, both provided by Sympatec (Clausthal-Zellerfeld, Germany), was used to this end. A powder mass of approximately 10 mg was placed on the RODOS dry disperser and dispersed at 3 bars. An R3 lens was fitted to the diffraction unit, enabling it to size particles between 0.1 and 175 μm. Powders were measured in triplicate. The average and standard deviation were calculated over six measurements after the data of the two duplicate amikacin batches were grouped together, or over nine measurements for the three isoniazid batches.

### 2.4. Automatic Filling

For the automatic filling of the SDCs an Omnidose drum filler (Harro Höfliger, Allmersbach im Tal, Germany) was used. For the isoniazid and amikacin powders, pellets were made with vacuum pressures of 200, 400, and 600 mbar. The emptying of the boreholes was done with a pressure of 0.5 bars, while the cleaning pressure was set at 1.8 bars. Per SDC, 24 pellets were dosed, calculated to result in a dose of around 55 mg, depending on the compound and the vacuum pressure. A row of eight SDCs was filled in approximately 35 s with this nominal dose. For isoniazid higher doses were also tested, with nominal doses of 85 and 105 mg.

For amikacin, the effect of relative humidity (RH) during filling was also tested. A cover plate was made, which sealed the hopper of the Omnidose. The hopper was purged with pressurized air that was dried by leading it through a column filled with silica beads. Environmental conditions during amikacin filling were a temperature between 20.4 and 22.5 °C and an RH between 34 and 40%.

### 2.5. Uniformity of Dosage Units

To determine the reproducibility of the automatic filling process, the uniformity of dosage units was determined according to procedure 2.9.47—Demonstration of uniformity of dosage units using large sample sizes—of the European pharmacopoeia 9.0. Based on the decision table in procedure 2.9.40, it was decided that the analysis could be performed based on mass variation (MV), as it was deemed that the different components after spray-drying have a similar uniform distribution through the powder as “solutions freeze-dried in final container”. Moreover, the drug content in the powders was far over 25%, with 99% for amikacin and 97% for isoniazid.

### 2.6. Dynamic Vapor Sorption

Dynamic vapor sorption (DVS) analysis was performed on the amikacin formulation using a DVS-1 (Surface Measurement Systems, Middlesex, United Kingdom). About 35 mg of sample was used for each run. Two different runs were made. Both runs started at 0% RH and increased the RH when the mass change per time interval (dm/dt) was below 0.0005%/min. During the first type of run, the RH was increased in steps of 10% to a maximum of 90%, after which the RH was decreased back to 0% RH with the same steps. During the second type of run the RH was increased to 40% in one step and was decreased back to 0% after that.

### 2.7. Moisture Content Determination

The moisture content of the amikacin pellets after automatic filling was obtained by Karl Fisher titration. An 831 KF Metrohm Coulometer (Herisau, Switzerland), equipped with a 728 stirrer, was used to this end. The reaction vessel of the coulometer contained Hydranal Coulomat AG, provided by Fluka (Seelze, Germany). Approximately 10 mg of pellets was dissolved in around 2 mL of Hydranal Coulomat AG, 1 mL of which was injected into the reaction vessel. The preparation of the samples was performed under dry nitrogen gas. Fifteen amikacin SDCs filled at 400 mbar were analyzed in duplicate. Five made at the start of the automatic filling run, five made at the end of the run, and five made at the end of the run under dried air (instead of dry nitrogen). The 400 mbar runs took approximately 4 h each. Moisture content is expressed as a percentage of the total powder mass (% *w/w*).

### 2.8. Scanning Electron Microscopy

Scanning electron microscopy (SEM) images were taken with the JSM 6460 SEM, (JEOL, Tokyo, Japan). Samples were fixed to an aluminum sample stub by the use of double-sided carbon tape, after which 10 nm of gold was sputter-coated by a JFC-1300 auto fine coater, also from JEOL. The coater was purged with argon gas. Images were taken with a spot size of 25 and an acceleration voltage of 10 kV under high vacuum, with a working distance of 10 mm. The secondary electron detector was used to construct the images.

### 2.9. Inhaler Dispersion Analysis

The dispersion behavior of the powders and the pellets from the Cyclops^®^ inhaler was determined with a HELOS diffraction unit equipped with an INHALER2000 adaptor (Sympatec, Clausthal-Zellerfeld, Germany). The diffraction unit was equipped with an R3 lens with a measuring range of 0.1 and 175 μm. The dispersion pressures tested were 2, 4, and 6 kPa. At each dispersion pressure, the powder and the 200, 400, and 600 mbar pellets were measured five times. The retention of the powder by the inhaler (i.e., the non-emitted powder mass) was determined gravimetrically. The FPF was calculated as a percentage of the emitted dose. The fine particle dose (FPD) was calculated by multiplying the FPF with the emitted dose (= FPF × (1 − inhaler retention)). For amikacin samples, prior to dispersion analysis approximately 20 mg of sweeper lactose in a size fraction of 250 to 315 μm was added manually to the SDC.

### 2.10. High-Speed Imaging

The dispersion of the powders and pellets in the Cyclops^®^ inhaler were imaged during the inhaler dispersion analysis with a Phantom VEO 310L high-speed camera, supplied by Vision Research (Wayne, NJ, USA). The camera was equipped with an AF Zoom-Nikkor 24–85 mm f/2.8–4D IF lens, (Nikon, Tokyo, Japan). Light was provided by a MULTILED QT, supplied by GS Vitec (Bad Soden Salmünster, Germany). Images were taken at a rate of 3200 frames per second (fps), with a resolution of 1280 × 800 and an exposure time of 200 μs. Afterwards, each video was analyzed as follows: frame numbers were determined for the occurrence or completion of different events, such as the start of the inhaler dispersion analysis, complete emptying of the SDC, dispersion of the bulk of the powder, and dispersion of all the powder. Dispersion of the bulk was estimated visually when around 95–99% of the powder had left the air classifier of the Cyclops^®^. The difference in frame number between the start of the analysis and the three other events was determined and divided by 3200. This gives the duration of each event in seconds. The average and standard deviation was then calculated (*n* = 5).

### 2.11. Large SDC

To increase the nominal dose above what could physically fit in the standard SDC (350 mm^3^), an SDC of increased width was tested (520 mm^3^). The L-SDC is made out of polycarbonate, while the standard SDC is made out of low-density polyethylene. The larger SDC (L-SDC) was hand filled with 400 mbar isoniazid pellets from automatically filled standard SDCs to a nominal dose of 150 mg, the maximum that could physically fit.

### 2.12. Drop Test

To test the effect of the repeated dropping of the inhaler filled with either loose powder or pellets, sealed SDCs were placed in a test sieve on an AS200 “g” vibratory sieve shaker, provided by Retsch (Haan, Germany). SDCs were vibrated at 1.5 g for 30 s, after which inhaler performance (dispersion at 2, 4, and 6 kPa) was determined. The tested SDCs contained loose powder, or pellets filled at either 400 or 600 mbar of isoniazid or amikacin.

### 2.13. X-ray Diffraction Analysis

X-ray analysis was performed with a Bruker D2 Phaser (Billerica, MA, United States). Samples were scanned in a range of 5 to 60° 2θ with steps of 0.004° 2θ, each step taking 1 s. The air scatter screen and divergence slit openings were 3 mm and 1 mm, the detector opening was 5°. The sample stage was spun at 60 rpm. Two samples were measured—an amikacin 1% L-leucine sample stored at 0% RH and an amikacin 1% L-leucine sample stored at approximately 80% RH for one week.

## 3. Results

Three isoniazid and two amikacin batches were spray-dried. The fraction ≤5 µm for the isoniazid batches is 91.80 ± 3.55% and for amikacin 87.37 ± 1.35%. The yields were between 86% and 88% for the isoniazid batches and between 84% and 86% for the amikacin ones. The outlet temperature was approximately 26 °C during the spray-drying of isoniazid, and approximately 66 °C during the spray-drying of amikacin. 

In Table 1, the results of the uniformity of dosage units test for the isoniazid formulation after filling SDCs at different vacuum pressures are given. According to the pharmacopeial procedures described in the material and methods section, the acceptance value should be ≤15. As can be seen, at all three vacuum pressures, the acceptance value is considerably lower. Furthermore, none of the specification results were obtained. Lastly, as expected, the average dose increases with increasing vacuum pressure.

The results of the uniformity of dosage units tests for the amikacin powder are shown in Table 2. The SDCs filled at 200 and 600 mbar vacuum pressures have acceptance values below 15 and zero dosage units were out of specification. The 400 mbar pellets, made at the end of the day, have two out of specification results, where only one is allowed. During this run, sometimes empty or half-filled boreholes were observed, as well as aggregates in the powder bed. The 400 mbar pellets made under dry air seem more reproducible, with an acceptance values below 15 and zero out of specification dosage units. When looking at the 200 and 600 mbar pellets, the dosage increases with increasing vacuum pressure, as is expected. The 400 mbar pellets produced under dry air are made under different humidity conditions and thus cannot be directly compared.

**Figure 2:**
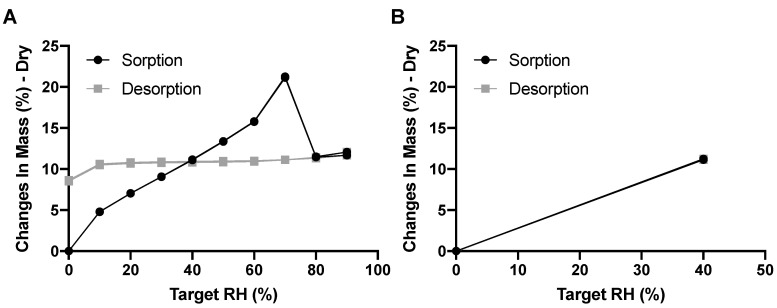
The DVS results for the amikacin formulation—panel (**A**) shows the sorption and desorption data from 0% to 90% RH and back in steps of 10% RH; panel (**B**) shows the data from 0% to 40% RH and back in steps of 40% RH (*n* = 2, both replicates shown).

Amikacin is known to be hygroscopic—the DVS results for the amikacin formulation confirms this (Figure 2). The amikacin formulation increases 4.81% in mass in the RH step of 0 to 10% RH. At 30% RH, just slightly below the RH experienced during automatic filling, the mass increased by 9.08%. It is interesting to note that mass increased up to 70% RH, after which the mass decreased. This is likely the result of moisture-induced crystallization [16]. During this process, powder fuses together, which was seen after the DVS measurement. This lowers the total surface area, and thus decreases the amount of adsorbed moisture. Moisture is likely absorbed in the crystalline structure, as the mass hardly decreases during the desorption phase. This is corroborated in Figure 2B as, when exposed to a maximum of 40% RH, no moisture was taken up permanently and no powder fusion was seen. The adsorption of moisture by amikacin is fast. When exposed to 40% RH, mass increased, by 8.07% in 33 and 44 min for the two duplicates, respectively.

**Figure 3:**
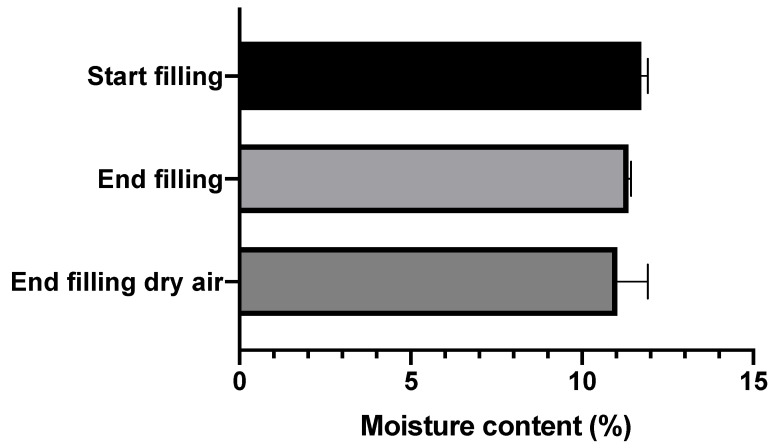
The moisture contents for the amikacin formulation automatically filled into SDCs. Start filling samples are the first five SDCs filled under normal conditions. End filling samples are the last five SDCs filled under normal conditions. The end filling dry air samples are the last five SDCs filled when automatic filling took place under dry air (average ± SD, *n* = 10).

The moisture contents of the automatically filled amikacin SDCs are shown in Figure 3. As can be seen, the moisture contents hardly differ between the start and end of the filling process, or between filling during normal and dry air conditions. All three groups fall between 11 and 11.5% moisture content.

**Figure 4:**
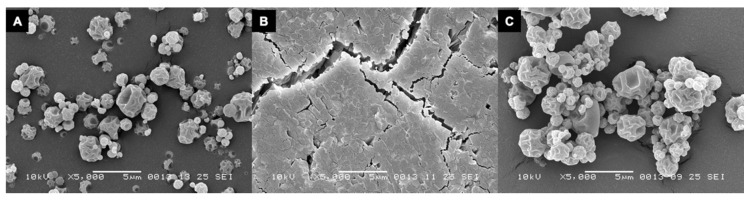
SEM images of the spray-dried amikacin formulation containing 1% L-leucine. Panel (**A**) shows the powder before DVS measurement. Panel (**B**) shows the same formulation after being exposed to 90% RH and panel (**C**) shows the same powder after being exposed to 40% RH during the DVS measurements. Magnification 5000× times.

The amikacin formulation after spray-drying displays a folded shell morphology, where the shell buckled as a result of internal voids, (Figure 4A). This morphology is completely lost after the DVS run to 90% RH and one huge aggregate is formed. The surface of this aggregate seems to consist of smaller particles fused together, as can be seen in Figure 4B. When exposed to a maximum of 40% RH, no fusion is seen and the folded shell morphology is retained.

**Figure 5:**
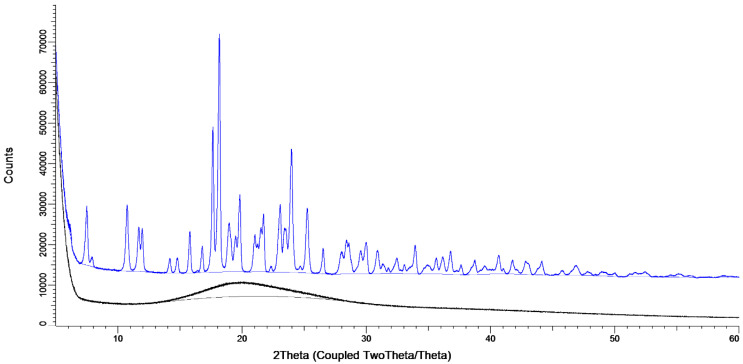
The X-ray spectra of the amikacin 1% L-leucine samples. The black line is the amikacin sample stored at 0% RH and the blue line is an amikacin sample stored at 80% RH for one week and is offset by 10,000 counts.

The X-ray diffraction data, shown in Figure 5, show the considerable effect of the storage conditions. When stored at 0% RH directly after spray, drying the sample is amorphous, evidenced by the presence of an amorphous halo and the lack of peaks (black line). When stored at 80% RH for one week, peaks are present, showing that a crystal structure has formed (blue line).

**Figure 6:**
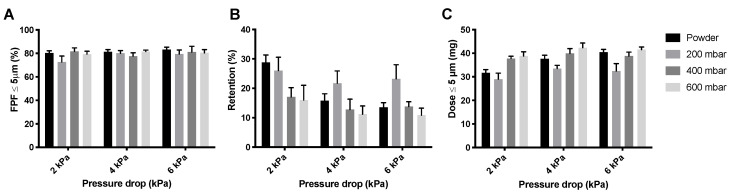
The effect of vacuum pressure on the dispersion from the Cyclops^®^ inhaler of 55 mg isoniazid. Panel (**A**) shows the FPFs for the loose powder and the three different pellets, panel (**B**) shows the retention, and panel (**C**) shows the FPD (average ± SD, *n* = 5). The FPD is calculated by multiplying the FPF with the emitted dose (= FPF × (1 − retention)).

Figure 6 shows the FPFs, the powder mass retained by the Cyclops^®^ inhaler, and the FPDs measured during the dispersion of the isoniazid formulation in its powder form as well as pellets. As can be seen, the FPFs (Figure 6A) do not seem to differ much between the different pressure drops or between the different pellets and loose powder. Inhaler retention, however, does differ substantially (Figure 6B). Retention seems to decrease with increasing pressure drop. Furthermore, at 4 and 6 kPa, the 200 mbar pellets have a higher retention than the loose powder and the 400 and 600 mbar pellets. At 2 kPa, the 400 and 600 mbar pellets have substantially lower retentions than the 200 mbar pellets and loose powder. This is reflected in the FPDs (Figure 6C), the 400 and 600 mbar pellets seem to result in higher FPDs than the 200 mbar pellets and loose powder, with the exception of the loose powder measured at 6 kPa.

**Figure 7:**
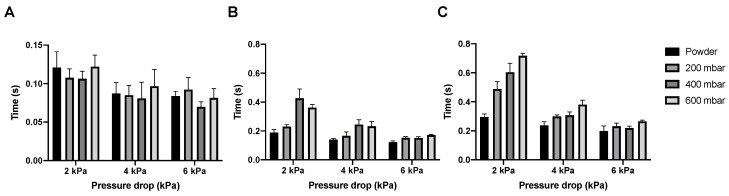
The dispersion times for 55 mg isoniazid. Panel (**A**) shows the time it took to empty the SDC, panel (**B**) shows the time it took for the bulk of the powder to be dispersed, and panel (**C**) shows the time needed to disperse the complete dose (average ± SD, *n* = 5).

In Figure 7 the dispersion times for the isoniazid powder and pellets are shown. The emptying of the SDC happens almost immediately for the powder and all different pellets, with the dose having left the SDC under 0.14 s at every condition tested. Bulk and total dose dispersion time depend on the pressure drop used, with dispersion being quicker at higher pressure drops, which is expected. However, whatever pressure drop is used, dispersion is fast. Bulk dispersion varies between 0.5 and 0.15 s and total dispersion between 0.75 and 0.21 s, depending on pressure drop and vacuum pressure.

**Figure 8:**
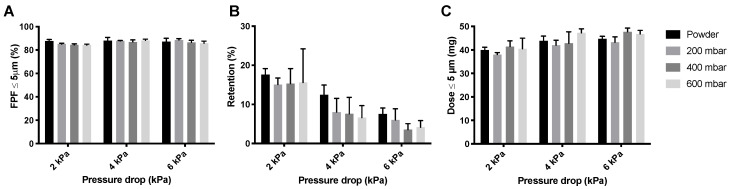
The effect of vacuum pressure on the dispersion of 57 mg amikacin. Panel (**A**) shows the FPFs found for the loose powder and three different pellets, panel (**B**) shows the inhaler powder retention, and panel (**C**) shows the FPD (average ± SD, *n* = 5). The FPD is calculated by multiplying the FPF with the emitted dose (= FPF × (1 − retention)).

The inhaler dispersion data for the amikacin loose powder and the three different pellets are shown in Figure 8. For the 400 mbar samples, the pellets made under dry air were used. The effect on the FPF, shown in Figure 8A, is small. However, retention (Figure 8B) seems to be lower for pellets than for the loose powder. This is further illustrated in the FPD (Figure 8C), where the dose is somewhat higher for the 400 and 600 mbar pellets at 2 and 6 kPa.

**Figure 9:**
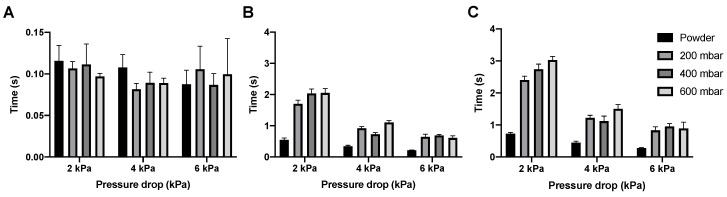
The dispersion times for 57 mg amikacin. Panel (**A**) shows the time it took to empty the SDC, panel (**B**) shows the time it took for the bulk of the powder to be dispersed, and panel (**C**) shows the time needed to disperse the complete dose (average ± SD, *n* = 5).

SDC emptying time for the amikacin formulation is similar to that of the isoniazid formulation, with the cartridge being emptied within 0.14 s (Figure 9A). Unlike isoniazid, the pelletization of the amikacin formulation substantially increases the bulk and total dispersion times, especially at 2 kPa (Figure 9B,C). Additionally, the difference between 2 kPa and the higher pressure drops is larger, with up to a second of difference between 2 and 4 kPa for the bulk dispersion of the 400 mbar pellets.

**Figure 10:**
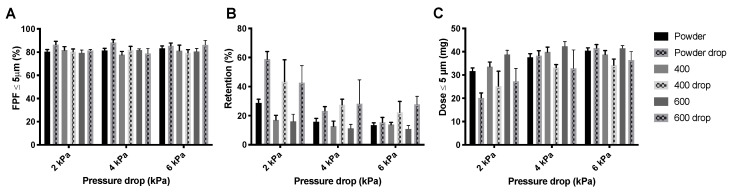
The effect of the drop test on the inhaler dispersion of 55 mg isoniazid. Panel (**A**) shows the FPFs for the powder, and the 400 and 600 mbar pellets before and after the drop test. Panel (**B**) shows the retention for these samples and panel (**C**) shows the FPD (average ± SD, *n* = 5). The FPD is calculated by multiplying the FPF with the emitted dose (= FPF × (1 − retention)).

The drop test does not seem to have a substantial effect on the FPF generated from isoniazid powder or pellets (Figure 10A). However, the powder retention is increased, especially at 2 kPa (Figure 10B). Visual observation showed that most pellets were disintegrated after the drop test and that the retention predominantly increased in the SDC, where the now loose powder was compressed against the wall and cover foil. The increase in retention is reflected in the FPD (Figure 10C), drop test pellet samples have lower FPDs.

**Figure 11:**
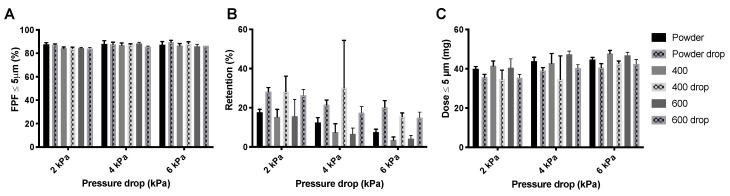
Effect of the drop test on the dispersion of 57 mg amikacin by the Cyclops^®^. Panel (**A**) shows the FPF of the different products before and after the drop test, panel (**B**) shows the retention and panel (**C**) shows the FPD (average ± SD, *n* = 5). The FPD is calculated by multiplying the FPF with the emitted dose (= FPF × (1 − retention)).

Similar to isoniazid, the FPF generated from the different amikacin products is not affected by the drop test (Figure 11A). Additionally, for this product the retention is increased by the drop test (Figure 11B). However, the increase in retention seems lower than that for the isoniazid pellets. Pellets were also not completely disintegrated. This is reflected in the FPD (Figure 11C). FPD is lower for the drop test samples, but this decrease is less substantial than for isoniazid.

**Figure 12:**
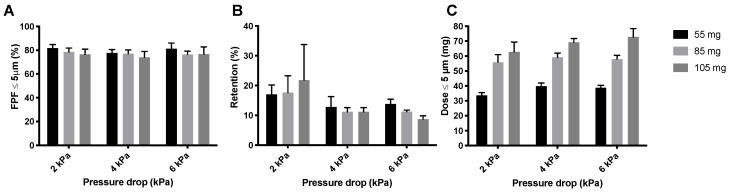
The inhaler dispersion results for three different nominal doses of isoniazid pellets produced at 400 mbar. Panel (**A**) shows the FPFs, panel (**B**) shows the inhaler retention and panel (**C**) shows the FPD (average ± SD, *n* = 5). The FPD is calculated by multiplying the FPF with the emitted dose (= FPF × (1 − retention)). The three different nominal doses used were, 55 mg (55.82 ± 0.25 mg), 85 mg (86.01 ± 0.72 mg), and 105 mg (105.30 ± 1.17 mg).

The compression of the powder that occurs during the automatic filling allowed for an increase in the mass that can be filled into the SDCs without decreasing dispersion efficiency. This is interesting because it may reduce the number of inhalations that is required for the administration of a complete dose. Increasing the nominal dose of isoniazid has a negligible effect on the FPF (Figure 12A), with a maximum decrease of 4% at 2 kPa with increasing dose. Retention increases with increasing dose at 2 kPa (Figure 12B), and decreases slightly with increasing nominal dose at 4 and 6 kPa. The FPD increases substantially by increasing the nominal dose (Figure 12C). From a FPD of 39.98 ± 2.07 mg, obtained from a nominal dose of 55 mg at 4 kPa to 69.23 ± 2.62 mg obtained from a nominal dose of 105 mg. The L-SDC results in a comparable FPD with a nominal dose of 105 mg. The maximum nominal dose that could fit in the L-SDC was 150 mg. The FPD is increased substantially, resulting in an average FPD of 107.35 ± 13.52 mg. It is interesting to note that the FPF of 70.90 ± 7.67% for the 150 mg dose is only 3% lower than for the 105 mg SDC samples.

## 4. Discussion

Our results show that isoniazid and amikacin can be precisely and accurately dosed with the Omnidose system in a vacuum pressure range from 200 to 600 mbar, as shown in Table 1 and Table 2. Powder flowability decreases with decreasing particle size, with particles for pulmonary administration showing poor flow characteristics [17]. However, the poor flowability, expected from the two tested formulations, do not seem to result in unreliable dosing.

Amikacin automatic filling needs to take place under a dry environment. As can be seen in Figure 2A, amikacin is hygroscopic, and between 70% and 80% RH a structural change in the powder was found to occur. A similar issue was seen for the isoniazid formulation, albeit between 80% and 90% RH [14]. This is likely moisture induced crystallization [16]. This is corroborated by the X-ray data (Figure 5). When stored at 80% RH, amikacin was crystalline, while it was amorphous when stored at 0% RH. The moisture-induced crystallization for amikacin is further corroborated by Figure 4, where the morphology of the spray-dried powder before and after DVS measurement to 90% RH is radically different. Before DVS, a typical folded-shell morphology is seen for spray dried material with L-leucine [18]. After DVS, a sizable aggregate is formed, which seems to consist of smaller particles fused together, likely a result of crystallization. However, based on Figure 2, the aggregate is formed between 70% and 80% RH. The automatic filling of amikacin took place between 34 and 40% RH. As can be seen in Figure 4C, no aggregates are formed when the formulation is exposed to 40% RH. This makes it unlikely that aggregates are formed during the filling process via this mechanism. The adsorption of water during the process and, as a result, the formation of capillary forces, which reduces powder flowability, might explain the amikacin results. Amikacin adsorbs moisture fast enough for this phenomenon to happen during automatic filling. However, as can be seen in Figure 3, moisture content at the start of the run and at the end are the same. Furthermore, the moisture content of the pellets produced under dry air are the same as well. While it is likely that capillary forces play a role, further research is needed to clarify this phenomenon.

Automatic filling improves the FPD of isoniazid (Figure 6). Isoniazid pellets made at vacuum pressures of 400 and 600 mbar result in lower retentions than the loose powder and 200 mbar pellets. FPF was similar, while an increase was expected. An increase in vacuum pressure likely increases the tensile strength of the pellets. Tensile strength for agglomerates is calculated by the following formula: σ = 15.6 ((φ^4^ × W)/d), where σ is the tensile strength, W is the work of cohesion between particles, d is the diameter of the particles, and φ is the packing fraction [19]. The packing fraction describes the density of a single pellet. As can be seen in Table 1, the nominal dose increases with higher vacuum pressures and an equal number of pellets, meaning that the density, and thus the packing fraction, increases. The resulting increase in packing fraction increases the tensile strength. This results in harder pellets, which are likely subject to stronger pellet-to-wall collision forces in the classifier of the Cyclops^®^, dispersing them more efficiently. However, this was not found to be the case. Retention depends on the applied vacuum pressure and the nominal dose (Figure 6B and Figure 12B). A higher vacuum pressure and a higher nominal dose both reduce the retention. The effect of the vacuum pressures may be explained by the increased tensile strength. As a pellet has a higher tensile strength, it does not disintegrate and compact against the SDC walls as easily, where most of the retention was observed. The effect of the nominal dose can be explained by the fact that, usually, the retention does not increase linearly with increasing nominal dose. The absolute amount retained by the inhaler is more or less the same, because it is partly determined by the surface of the inhaler, but with respect to percentage, this results in a decrease.

Just as for isoniazid, the automatic filling of amikacin improves its FPDs (Figure 8). Again, the 400 and 600 mbar pellets seem optimal. However, the differences between the loose amikacin powder and the pellets is smaller than it was for isoniazid. Amikacin is known to be very cohesive and adhesive [20]. It is likely that, as a result of these physicochemical properties, spontaneously formed aggregates may already occur in loose powder.

Dropping the inhaler frequently has a substantial impact on the FPD for isoniazid and amikacin (Figure 10 and Figure 11), with an increase in retention being seen. Visual inspection of the isoniazid SDCs showed that pellets had been largely destroyed and that powder had compacted against the sides and back of the SDC. This powder was not entrained in the airstream during the measurement, which decreases the FPD being delivered by the device. The supposedly higher tensile strength of the amikacin pellets may explain its lower retention after dropping than that of the isoniazid formulation. While, again, the retention by the SDC was increased following the test, pellets were destroyed to a smaller extent than for isoniazid. Some loose powder was still compacted against the walls of the SDC and not entrained. However, the difference here is less substantial than it was for isoniazid. It is important to note that the drop test is a worst-case scenario, with the pellets experiencing vibrations, which a disposable device is unlikely to experience in real-life use.

All isoniazid samples are dispersed in a very short time, leaving room to increase the nominal dose (Figure 7). At 4 kPa, the bulk of the 400 and 600 mbar pellets is dispersed in around 0.24 s. The Cyclops^®^ inhaler has an airflow rate of 46.2 L/min at 4 kPa, which means that the bulk of the powder is dispersed in the first 0.18 L of air. In general, to deliver powder deep into the lungs, powder has to be dispersed in the first 0.5 to 1.5 L of air [21]. The dispersion of isoniazid is substantially quicker and thus higher nominal doses could probably be dispersed within the first 0.5 to 1.5 L of air as well—the rationale being that an increase in nominal dose usually means an increase in dispersion time. Furthermore, as dispersion is so quick, it lowers the chance that the classifier of the Cyclops^®^ gets overloaded with powder or pellets, which could lower its efficiency (because of a crowding-out effect) and thus the FPF. In our previous study with loose powder, higher doses were tested as well [14]. Our findings were that a dose of 80 mg was optimal, which resulted in a FPD of 58.00 ± 2.56 mg. A nominal dose of 100 mg did not improve the FPD delivered by the Cyclops^®^. Here, as a result of automatic filling, the nominal doses tested were 85 and 105 mg. The 85 mg SDCs result in a FPD of 59.12 ± 2.87 mg at 4 kPa, similar to our previous results (Figure 12). What is unlike our previous results is that, with pellets, the nominal dose of 105 mg does increase the FPD to 69.23 ± 2.62 mg. Furthermore, with a dispersion time of 0.27 s the time has increased only slightly. Higher nominal doses are thus possible from a timing point of view. Nevertheless, a higher nominal dose does not physically fit in the SDC. To amend this, an SDC of increased width was designed and tested, named the L-SDC. The highest nominal dose that could fit in the L-SDC, which is 150 mg, was tested. Bulk dispersion took place in the first 0.39 L of air. FPF seems to lower only slightly with this increase in nominal dose, while the FPD increases to 107.35 ± 13.52 mg; however, the standard deviation is relatively high. During one of the five measurements half of the pellets got stuck in the L-SDC for around two seconds before emptying as well. This had the effect of gradually feeding the classifier of the Cyclops^®^, improving its efficiency. It might thus be of interest to design a SDC which always feeds the classifier gradually, as long as the powder is dispersed in the first 0.5 to 1.5 L of air. However, it is likely that there is a maximum FPD that can be administered in a single inhalation, after which cough and tolerability by the patient will become an issue. Future research is necessary to determine this maximum FPD.

The dispersion time is considerably longer for amikacin (Figure 9), with the bulk of the 400 mbar pellets being dispersed in 0.72 s and the 600 mbar pellets in 1.11 s, meaning that the bulk is dispersed in the first 0.6 to 0.9 L of air. As a result, no higher nominal doses were tested. The longer dispersion time might be a consequence of the higher tensile strength of the pellets. The classifier requires more time and pellet-to-wall collisions to completely disperse an amikacin pellet.

In future studies it might be of interest to determine the suitability of other automatic filling techniques for the automatic filling of the isoniazid and amikacin formulations, together with their effect on dispersibility and inhaler retention.

## 5. Conclusions

The isoniazid and amikacin formulations can be precisely and accurately dosed with the Omnidose system, with both products fulfilling the uniformity of dosage units requirements of the European pharmacopoeia. The Cyclops^®^ inhaler dispersed the produced pellets effectively, with the optimal vacuum pressure being in the range of 400 to 600 mbar for both products. The isoniazid pellets were dispersed quickly, which gave room for the nominal dose to be increased to 150 mg. The amikacin SDCs were kept at 57 mg because of the longer dispersion time required for this product. This resulted in maximum FPDs of 107.35 ± 13.52 mg for isoniazid and 47.26 ± 1.72 mg for amikacin. These results may contribute to the development and upscaling of production of an amikacin and an isoniazid dry powder product for inhalation. Furthermore, these findings may contribute to the understanding of the upscaling of high dose dry powder products for inhalation, which use a limited amount of excipient on a whole.

## Figures and Tables

**Table 1:** The results of the uniformity of dosage units test after dosing with the Omnidose for the isoniazid with 3% trileucine powder at three different vacuum pressures. Acceptance value (criteria ≤ 15) and the amount of dosage units out of specification are determined according to European pharmacopoeia 9.0, procedures 2.9.40. and 2.9.47.

Vacuum Pressure	Sample Size	Average Dose (mg)	Acceptance Value	Out of Specification
200 mbar	56	53.53 ± 0.69	1.37	0
400 mbar	223	55.82 ± 0.25	0.55	0
600 mbar	56	58.18 ± 0.68	1.36	0

**Table 2:** The results of the uniformity of dosage units tests after dosing with the Omnidose for the amikacin with 1% L-leucine powder at three different vacuum pressures. Acceptance value (criteria ≤ 15) and the amount of dosage units out of specification are determined according to European pharmacopoeia 9.0, procedures 2.9.40. and 2.9.47. * pellets produced under dry air.

Vacuum Pressure	Sample Size	Average Dose (mg)	Acceptance Value	Out of Specification
200 mbar	56	52.08 ± 0.75	1.50	0
400 mbar	223	51.81 ± 3.85	8.46	2
400 mbar *	166	57.01 ± 2.05	4.48	0
600 mbar	56	57.05 ± 0.97	1.95	0

## References

[1] Sibum I., Hagedoorn P., de Boer A.H., Frijlink H.W., Grasmeijer F. (2018). Challenges for pulmonary delivery of high powder doses. Int. J. Pharm..

[2] Labiris N.R., Dolovich M.B. (2003). Pulmonary drug delivery. Part I: Physiological factors affecting therapeutic effectiveness of aerosolized medications. Br. J. Clin. Pharmacol..

[3] Castellanos A. (2005). The Relationship between Attractive Interparticle Forces and Bulk Behaviour in Dry and Uncharged Fine Powders. Adv. Phys..

[4] Hersey J.A. (1975). Ordered mixing: A new concept in powder mixing practice. Powder Technol..

[5] Grasmeijer F., Grasmeijer N., Hagedoorn P., Boer H.W.F., de Bore A.H. (2015). Recent advances in the fundamental understanding of adhesive mixtures for inhalation. Curr. Pharm. Des..

[6] Edwards D. (2010). Applications of capsule dosing techniques for use in dry powder inhalers. Ther. Deliv..

[7] Faulhammer E., Fink M., Llusa M., Lawrence S.M., Biserni S., Calzolari V., Khinast J.G. (2014). Low-dose capsule filling of inhalation products: Critical material attributes and process parameters. Int. J. Pharm..

[8] Stranzinger S., Faulhammer E., Calzolari V., Biserni S., Dreu R., Šibanc R., Paudel A., Khinast J.G. (2017). The effect of material attributes and process parameters on the powder bed uniformity during a low-dose dosator capsule filling process. Int. J. Pharm..

[9] Newton J.M. (2012). Filling hard gelatin capsules by the dosator nozzle system—Is it possible to predict where the powder goes?. Int. J. Pharm..

[10] Llusa M., Faulhammer E., Biserni S., Calzolari V., Lawrence S., Bresciani M., Khinast J. (2014). The effects of powder compressibility, speed of capsule filling and pre-compression on plug densification. Int. J. Pharm..

[11] Llusa M., Faulhammer E., Biserni S., Calzolari V., Lawrence S., Bresciani M., Khinasta J. (2013). The effect of capsule-filling machine vibrations on average fill weight. Int. J. Pharm..

[12] Jones B.E. (2001). The filling of powders into two-piece hard capsules. Int. J. Pharm..

[13] Sibum I., Hagedoorn P., Frijlink H.W., Grasmeijer F. (2019). Characterization and Formulation of Isoniazid for High-Dose Dry Powder Inhalation. Pharmaceutics.

[14] Sibum I., Hagedoorn P., Kluitman M.P.G., Kloezen M., Frijlink H.W., Grasmeijer F. (2020). Dispersibility and Storage Stability Optimization of High Dose Isoniazid Dry Powder Inhalation Formulations with L-Leucine or Trileucine. Pharmaceutics.

[15] WHO (2018). Global Tuberculosis Report 2018.

[16] Zafar U., Vivacqua V., Calvert G., Ghadiri M., Cleaver J.A.S. (2017). A review of bulk powder caking. Powder Technol..

[17] Tay J.Y.S., Liew C.V., Heng P.W.S. (2017). Powder Flow Testing: Judicious Choice of Test Methods. AAPS PharmSciTech.

[18] Vehring R. (2008). Pharmaceutical particle engineering via spray drying. Pharm. Res..

[19] Das S.C., Behara S.R.B., Bulitta J.B., Morton D.A.V., Larson I., Stewart P.J. (2012). Powder strength distributions for understanding de-Agglomeration of lactose powders. Pharm. Res..

[20] Hoppentocht M., Akkerman O.W., Hagedoorn P., Frijlink H.W., De Boer A.H. (2015). The Cyclops for pulmonary delivery of aminoglycosides; A new member of the Twincer family. Eur. J. Pharm. Biopharm..

[21] Hoppentocht M., Hagedoorn P., Frijlink H.W., de Boer A.H. (2014). Technological and practical challenges of dry powder inhalers and formulations. Adv. Drug Deliv. Rev..

